# Deciphering the microbial diversity associated with healthy and wilted *Paeonia suffruticosa* rhizosphere soil

**DOI:** 10.3389/fmicb.2022.967601

**Published:** 2022-08-17

**Authors:** Manman Jia, Xin Sun, Man Chen, Shuang Liu, Jinxing Zhou, Xiawei Peng

**Affiliations:** ^1^College of Biological Sciences and Technology, Beijing Forestry University, Beijing, China; ^2^Jianshui Research Station, School of Soil and Water Conservation, Beijing Forestry University, Beijing, China; ^3^Beijing Key Laboratory of Food Processing and Safety in Forestry, Beijing Forestry University, Beijing, China; ^4^Institute of Tree Development and Genome Editing, Beijing Forestry University, Beijing, China

**Keywords:** *Paeonia suffruticosa*, root rot, microbial network, plant pathogen, rhizosphere microbial community

## Abstract

Plant health is closely related to the soil, where microorganisms play a critical and unique role. For instance, *Paeonia suffruticosa* is an emerging woody oil crop in China with attractive development and utilization prospects. However, black root rot causes wilting of the aboveground plant parts, which significantly affected its seed yield and quality. Studies found that soil microorganisms are critical in maintaining plant health, but how changes in the soil microbial communities affect the healthy and diseased oil peony is unclear. Therefore, our present study used high throughput sequencing and BIOLOG to analyze the rhizosphere soil microbial communities of healthy and diseased oil peonies. Our results revealed that the physical and chemical properties of the soil of the diseased plants had changed, with the ability to metabolize the carbon source being enhanced. Moreover, our research highlighted that the oil peony-infecting fungal pathogenic genus (*Fusarium*, *Cylindrocarpon*, and *Neocosmospora*) was closely associated with oil peony yield reduction and disease aggravation. Further network analysis demonstrated that the bacterial and fungal networks of the diseased plants were more complex than those of the healthy plants. Finally, the inter-kingdom network among the diseased plants further indicated that the lesions destroyed the network and increased the intraspecific correlation between the fungal groups.

## Introduction

Oil peony, a unique new woody oil crop in China, is among the world’s top woody oil tree species, which includes olive, *Camellia oleifera*, and oil palm. The peony seed oil contains up to 92.26% unsaturated fatty acids, with over 42% α-linolenic acid, also known as “blood nutrients” (Cheffi et al., 2019). The *Fusarium* species complex often causes root rot, a classical soil-borne disease that attacks many economically important crops, including potato and camellia (Kendrick, 2003; Dugan et al., 2010; Hernandez et al., 2021). Root rot in oil peony is caused by *Fusarium solani.* At the disease onset, the pathogen enters the plant through the roots, then expands to the medulla, followed by the scattered fleshy roots, and even infects the entire root, thereby finally causing severe production losses annually.

Various soil biochemical factors affect the growth and survival of soil pathogens and the plant’s nutrient availability. Among them, soil microorganisms are crucial factors for plants and pathogens, since they are critical in regulating soil fertility, nutrient cycling, promoting plant health, and protecting plants from diseases (Lauber et al., 2008; Faoro et al., 2010; Rousk et al., 2010). Microbiota associated with the host plant, especially their roots, determines the soil-borne pathogen’s infection capability. Thus, assembling a self-serving rhizosphere microbiota is vital for both plants and pathogens. Plants recruit beneficial microbes to stimulate plant growth, elicit plant systemic defense, and attack pathogens. When pathogens invade plants and cause diseases, the original rhizosphere microecological balance is disturbed, subsequently changing the soil properties, microbial community structure, and metabolic functions (Li et al., 2010; Jaffuel et al., 2016; Mueller et al., 2016). Microbial community composition is primarily influenced by the cooperative and competitive interactions among the numerous microbial members that help maintain plant health (van der Heijden et al., 2016; Duran et al., 2018). Currently, the co-occurrence networks are widely used in inferring potential microbial interactions (De et al., 2018; Hernandez et al., 2021). The uniformity and complexity of the networks have been shown to be critical for stable host–microbial interactions (Wang et al., 2021).

Such an analysis is vital, as a balanced microbiome is crucial for the health of humans, plants, and the environment, while diseases are often associated with microbial dysbiosis (Hooks et al., 2017). Compared to healthy individuals, communities with microbial imbalances have more significant differences in their composition. Additionally, high microbial diversity and stable community composition have significant effects on the prevention of pathogen invasion and establishment (Ning et al., 2011), counteract the pathogen growth (Yuan et al., 2012), competition with pathogens for nutrients (Weller et al., 2002), direct promotion of plant growth (Langfelder and Horvath, 2007), and modulation of host immunity. Therefore, understanding the soil microbial composition of the rhizosphere is the key to understanding the spread of soil-borne diseases. Nevertheless, our current understanding of the potential interactions within the complex plant-associated microbiomes and their response to pathogen invasion still remains limited.

Therefore, this study evaluated the characteristics and differences between the diseased and healthy plants, including the soil’s physicochemical properties and the microbial community. In this study, we hypothesized: (1) soil properties are correlated with the soil microbial community and root rot, and (2) soil microbial community is altered in the root rot infected soils. For this purpose, we employed the BIOLOG and Illumina Miseq high-throughput sequencing technologies to compare the rhizosphere soil microbial communities of the healthy and diseased oil peony plants in different regions, thus providing theoretical support for the biological control of the oil peony root rot disease.

## Materials and methods

### Sample selection and physiochemical soil properties

All samples were collected from the main oil peony plant bases in Shanxi Province, China, at Wuxiang (112°51′56″ E, 36°50′40″ N), Xiangyuan (11258′15″ E, 36°27′42″ N), and Huguan (113°11′56″ E, 36°7′24″ N) (Supplementary Figure 1). The three oil peony planting bases were empty farmland before the planting of oil peony began in the last five years. Fertilization, weeding, and other daily management measures remained the same. The oil peony cultivar used was *P. suffruticosa*, and mature peonies were sampled at all sites. At each site, healthy plants not only referred to the above-ground plant growth being good and consistent without leaf wilting but also having an underground fleshy root surface without black spots. Accordingly, the diseased plants referred to the noticeable wilting and death of the aboveground parts, i.e., yellowing and wilting of the leaves, along with the blackening of the underground fleshy roots. Three replicates of each of the healthy and diseased plants were collected from three different plots (20 m × 20 m) at each site. From each plot, soil samples were randomly collected using the five-point sampling method. Soils of the same type collected from the same plot were thoroughly mixed into one sample. Rhizosphere soil (defined as the soil that adheres to the root) was collected from the roots through manual shaking. Soil samples were put into labeled self-sealing bags and transported to the laboratory to determine their physical and chemical properties. Each soil sample was ground, sieved through a 2-mm sieve, and divided into three portions for further processing, i.e., stored in a 4°C refrigerator for BIOLOG, air-dried for chemical property analysis, and stored at −80°C for DNA extraction. Samples were marked with letters and numbers as follows: H1: healthy plants in Wuxiang; D1: diseased plants in Wuxiang; H2: healthy plants in Xiangyuan; D2: diseased plants in Xiangyuan; H3: healthy plants in Huguan; D3: diseased plants in Huguan.

### Physical and chemical properties of soil

The soil’s physicochemical parameters were measured according to the oven dry-weight method, which helped estimate the soil moisture content. Then soil pH was determined using a glass electrode meter (Sartorius PB-10) in a suspension of 1 g of soil in 5mL of distilled water. Available P (AP) was extracted using sodium bicarbonate and subsequently measured by the molybdenum blue method. The available K (AK) was determined by flame photometry (Wu et al., 2016; Xia et al., 2016), while the available N (AN) was determined using potassium persulfate oxidation. The organic matter (OM) content was determined as described previously (Inclán et al., 2015; Restrepo et al., 2015).

### DNA extraction, polymerase chain reaction amplification, sequencing analysis

Total genomic DNA was extracted directly from these samples using the FastDNA^®^ spin kit according to the manufacturer’s protocol (MP Bio, Santa Ana, United States). DNA concentrations were then determined using a NanoDrop ND-2000 UV–vis spectrophotometer (Thermo Scientific, Wilmington, United States). The DNA’s integrity was assessed *via* 1% agarose gel electrophoresis. The bacterial 16S rRNA gene’s V3-V4 hypervariable regions were amplified using the 341F (5′-CCTAYGGGRBGCASCAG-3′) and 806R (5′-GGACTACNNGGGTATCTAAT-3′) primers. To amplify the fungal ITS sequences, the primers ITS1-F (5′-GGAAGTAAAAGT CGTAACAAGG-3′) and ITS1-R (5′-GCTGCGTTCTTCATCGATGC-3′) were used. Polymerase chain reaction (PCR) reaction system was as follows: Phusion Master Mix (2×) 15 μl; Primer (2 μm) 3 μL; 10 μl template DNA; H_2_O 2 μL, total 30 μl. Reaction procedure: pre-denaturation at 98 °C for 1 min; The 30 cycles included (98 °C, 10 s; 50 °C, 30 s; 72 °C, 30 s); It was then extended for 5 min at 72 °C. 16S rRNA and ITS rRNA tag-encoded high-throughput sequencing were carried out on the Illumina MiSeq platform (Novogene, Beijing, China). Pairs of reads from the original DNA fragments were merged based on the previously described method, with sequencing reads being assigned to each sample according to the unique barcode. Sequences were analyzed through the QIIME software package (Quantitative Insights into Microbial Ecology) and the UPARSE pipeline. The reads were first filtered using the QIIME quality filters while using the default settings for Illumina processing in QIIME. Then the UPARSE pipeline was utilized to detect the operational taxonomic units (OTUs) at 97% similarity. For each OTU, a representative sequence was selected and used to assign taxonomic composition employing the RDP classifier.

### Microbial metabolic function

The functional diversity of the soil microbial communities was estimated using the BIOLOG method. EcoPlates were used for the BIOLOG assay to determine the microbial carbon source utilization profile. One gram of fresh rhizosphere soil from around diseased or healthy plants was mixed with 99 ml of 0.85% sterile NaCl solution, then shaken for 30 min on a reciprocal shaker. Then, 150 μL of the solution was added into each well of the EcoPlate with 31 carbon sources and incubated in darkness at 25°C. Plates were read every 24 h at 590 nm with a total time of up to 168 h. Average chromogenic development (AWCD) was used to evaluate the carbon source utilization capacity of microbial communities in the rhizosphere soil samples of healthy and diseased plants. The utilization of 31 carbon sources by the microbes in each sample was measured as described previously, and a principal coordinate analysis (PCoA) was used to detect the time-course of substrate utilization in healthy and diseased rhizosphere samples.

### Statistical analyses

The original data obtained by sequencing were spliced and filtered to obtain valuable clustered data. The operational taxonomic unit (OTU) was clustered under 97% similarity to calculate the number, annotate species, and obtain taxonomic information. The Alpha diversity indices (Chao1 index, Shannon index, and Simpson index) and the Goods-coverage index were calculated using Qiime software, while the taxonomic differences between the groups were compared utilizing the least-significant-difference (LSD) test with *t*-test adjustment. The statistical significance was *p* = 0.05 and heat maps were generated using custom R (version 3.2.5). Redundancy analysis (RDA) using the vegan package R (version 3.2.5) analyzed the relationships between microbial community structure, microbial species, and environmental variables.

### Network analysis

A network of OTU levels between the fungi and bacteria was constructed using rhizosphere microorganisms from both healthy and diseased plants to evaluate the complexity of the interactions between microbial taxa. Using the Spearman correlation analysis (SparCC), a tool for estimating correlation values from component data, the SparCC correlation with statistical significance (*P* > 0.05) was included in the network analysis. Nodes in the reconstructed network represent gate-level classification units, while edges represent significant positive or negative correlations between the nodes. The corresponding network graph relies on node number, edge number, modularity, community number, average node connectivity, average path length, diameter, and cumulative degree distribution. The data co-occurrence analysis used the psych package in R 3.5.2, while visualization and attribute measurement employed the Gephi computing network. The hub taxa per network were identified as within and among module connectivity, while the network stability was measured by the proportion of negative or positive correlations and modularity.

## Results

### Physical and chemical properties of soil

The physical and chemical properties of the soil are presented in Table 1. The ANOVA analysis revealed a difference between the healthy and diseased soils, where the soil’s AK, AP, and OM decreased significantly in the diseased soils as compared to the healthy soils. AN also showed a decreasing trend but with no significant difference. Compared with the healthy soil, pH and water content did not show any trend changes. Finally, the correlation analysis highlighted that AK was significantly positively correlated with OM (0.82) and AP (0.66) (Supplementary Figure 2).

**TABLE 1:** Physicochemical parameters of the soil samples.

Samples	Available N/mg⋅kg^–1^	Available P/mg⋅kg^–1^	Available K/mg⋅kg^–1^	Organic matter/g⋅kg^–1^	pH	Water content/%
H1	33.95 ± 1.98a	17.84 ± 2.41b	392.59 ± 11.75a	21.80 ± 0.90a	7.74 ± 0.02bc	13.34 ± 0.28b
D1	31.07 ± 1.44ab	11.01 ± 0.42c	335.61 ± 8.12b	19.13 ± 0.58b	7.68 ± 0.03c	14.05 ± 0.13a
H2	27.6 ± 0.91b	26.00 ± 1.22a	335.6 ± 23.46b	18.98 ± 0.77b	7.83 ± 0.05ab	12.47 ± 0.12c
D2	31.01 ± 1.54ab	11.04 ± 0.75c	243.87 ± 9.43c	15.68 ± 0.47c	7.9 ± 0.02a	11.81 ± 0.13d
H3	28.95 ± 1.36b	23.74 ± 0.93a	372.24 ± 7.29ab	16.63 ± 0.76c	7.8 ± 0.02b	10.85 ± 0.07f
D3	29.34 ± 1.44b	9.33 ± 0.45c	224.48 ± 21.25c	13.34 ± 0.65d	7.9 ± 0.05a	11.37 ± 0.15e

All data are presented as the mean ± SE. Different lowercase letters in the same column indicate significant (*p* < 0.05) differences between the healthy and diseased soils. H1, healthy plants in Wuxiang; D1, diseased plants in Wuxiang; H2, healthy plants in Xiangyuan; D2, diseased plants in Xiangyuan; H3, healthy plants in Huguan; D3, diseased plants in Huguan.

### Soil microbial diversity

**FIGURE 1:**
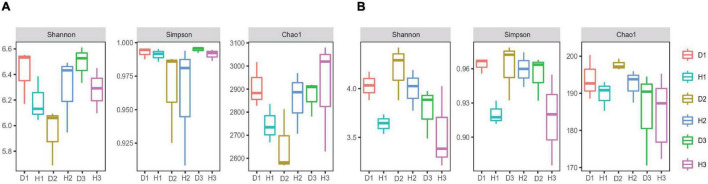
Microbial alpha diversity. The α-diversity indices of soil bacterial **(A)** and fungal **(B)** communities in the soils of healthy and diseased oil peony, Chao1 index for calculating bacterial abundance, Shannon index, and Simpson index for calculating bacterial diversity, respectively.

We assigned all raw sequence data to each sample based on their barcode sequence. We obtained 1184901 bacterial 16S rRNA and 1270956 fungal ITS high-quality reads from 18 samples, sorted into 3904 and 2872 bacterial fungal OTUs. The rarefaction curve indicated that most microorganisms were detected in the samples. We used the Shannon, Simpson, and Chao 1 indices to evaluate and compare the diversity and richness of bacterial communities among different soil samples (Figure 1). The results revealed no significant difference between the healthy and diseased soil. However, the differences in the soil’s microbial diversity at different sampling points were evident, with the overall effect of the diseased soil on microbial diversity and richness being non-significant.

### Soil microbial community composition and structure analysis

When grouping the OTUs at the phylum level of bacteria, we found that the diseased plants had more bacterial phyla, whereas healthy plants had a greater number of fungal phyla, with no significant differences between the diseased and healthy soils (Supplementary Figure 3). Proteobacteria, Actinobacteria, Acidobacteria, Bacteroidetes, Chloroflexi, and Firmicutes were the dominant phyla existing in all the samples, with Proteobacteria being the most dominant phylum accounting for 45.36% (H1), 43.12% (H2), and 28.49% (H3) in the healthy soils and 40.73% (D1), 44.13% (D2), and 51.38% (D3) in the diseased soils. Similarly, we observed no significant difference among the fungi at the phylum level between the healthy and diseased soils. For the fungi, Ascomycetes, Balloonomycetes, Basidiomycetes, and Mortierella, were the dominant phyla across all samples, with Ascomycota being the most dominant phylum accounting for 24.80% (H1), 25.82% (H2), and 41.45% (H3) in healthy soils and 38.49% (D1), 32.34% (D2), and 46.31% (D3) in diseased soils.

**FIGURE 2:**
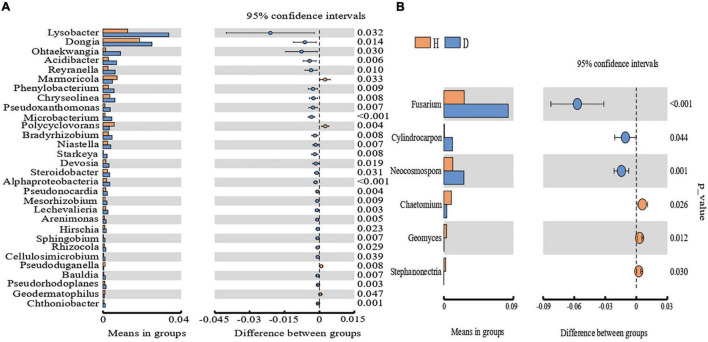
The difference in the relative abundance of bacteria **(A)** and fungi **(B)** between healthy and diseased plants was calculated, and the *t*-test was used to calculate the *P*-value (*P* < 0.05). H and D refer to healthy and diseased plant soil.

We further explored the microbial community at the genus level and found differences in the microbial community structure in the healthy and diseased soils from different regions through the *t*-test (Figure 2). The abundances of thirty bacterial genera and six fungal genera were significantly different between the healthy and diseased soils (*p* < 0.05), as indicated by the *t*-test. Twenty-six bacterial genera were more abundant in the diseased soils, with bacterial genera like *Marmoricola, Polycyclovorans, Pseudoduganella*, and *Geodermatophilus* being more abundant in the healthy soils. Moreover, fungal genera like *Chaetomium, Geomyces*, and *Stephanonectria* were abundant in the healthy soil, while *Fusarium, Cylindrocarpon, Neocosmospora* were abundant in the diseased soil. Among them, *Fusarium* showed significant differences between diseased and healthy plant soils (*p* < 0.01).

### Relationship between microbial community and environmental variables

**FIGURE 3:**
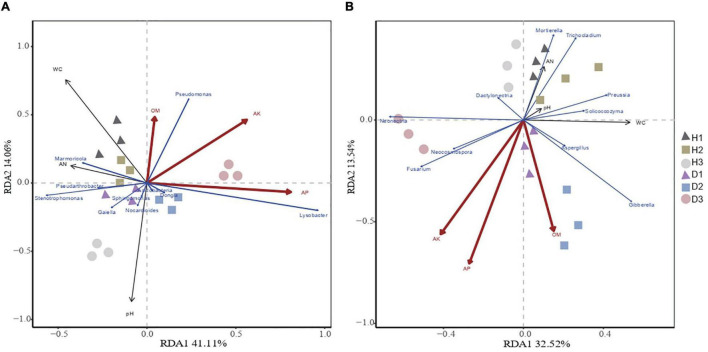
Redundancy analysis of abundant bacterial **(A)** and fungal **(B)** genus and soil properties for healthy and diseased samples from three sampling areas. The“H1,” “H2,” and “H3” refer to the three healthy soils. The “D1,” “D2,” and “D3” refer to the three diseased soils, respectively.

Redundancy analysis showed that the microbial community structure was affected by the plants’ health and soil properties (Figure 3). The microbial community structure in H1, H2, and H3 was correlated with AK, AP, and OM, while the microbial community structure in D1, D2, and D3 was correlated with pH. The redundancy analysis highlighted that the first and second components explained 35.74% and 26.85% of the total bacterial and total fungal variations, respectively. In the bacterial communities, the soil OM, AP, and AK were positively correlated with *Pseudarthrobacter*, *Marmoricola*, and *Pseudomonas* and were negatively correlated with *Dongia, Sphingomonas Acidobacteria*, *Gaiella*, *Nocardioides*, *Lysobacter*, and *Nocardioides.* With respect to the fungal communities, the soil OM, AP, and AK were positively correlated with *Trichocladium*, *Mortierella*, *Aspergillus, Solicoccozyma*, and *Preussia*, while being negatively correlated with *Fusarium*, *Gibberella*, *Dactylonectria*, *Neonectria*, and *Neocosmospora*. Thus, the soil properties, especially the available nutrients, highly influence the community structure of soil microbes.

### Microbial metabolic function

**FIGURE 4:**
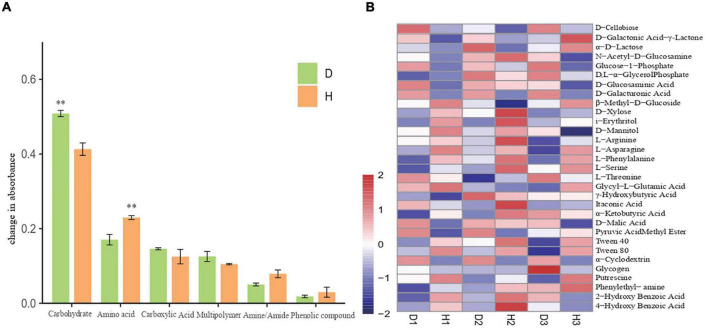
Six groups carbon source utilization rates of soil microorganisms of healthy and diseased oil peony **(A)**. Differences in utilization of 31 carbon sources by soil microorganisms **(B)**. The “D1,” “D2,” and “D3” refer to the three diseased soils, respectively.

We also analyzed the average absorbance of soil microorganisms and found that the carbon source utilization rate in the samples increased with time, with the carbon source targeted metabolic ability of diseased plants being higher than that of the healthy plants. After principal component analysis (PCA) of the AWCD value of the soil samples, the microbial flora of healthy and diseased soils showed a separated state of carbon substrate utilization, thus indicating the differences in microbial communities between the healthy and diseased soil samples. Upon comparing the utilization rates of six different functional groups (carbohydrates, amino acids, carboxylic acids, multipolymer, amines/amides, and phenolic compounds) by microorganisms (Figure 4A), we found that the utilization rate of carbohydrates by microbial communities in diseased soil was significantly higher than those in healthy soil. Additionally, although the amino acid utilization rate in soil microbial communities increased, there was no significant difference in the utilization rate of the other carbon sources. Furthermore, the thermal diagram (Figure 4B) indicates that in all samples, the substrate D-Xylose, i-Erythritol, L-Arginine, L-Asparagine, L-Phenylalanine, L-Serine, Tween 40, Tween 80, Phenylethyl-amine, and Putrescine were metabolized rapidly in diseased soil. The substrates, including D-Cellobiose, Glucose-1-Phosphate, D-Glucosaminic, D-Galacturonic, D-Malic Acid, and α−Cyclodextrin presented high utilization rates in healthy soils. The significant differences in the carbon substrate utilization patterns between the healthy and diseased soil communities involved L-Asparagine and phenylethyl-amine, with high and low utilization in the diseased and healthy samples, respectively.

### Co-occurrence network analysis

**FIGURE 5:**
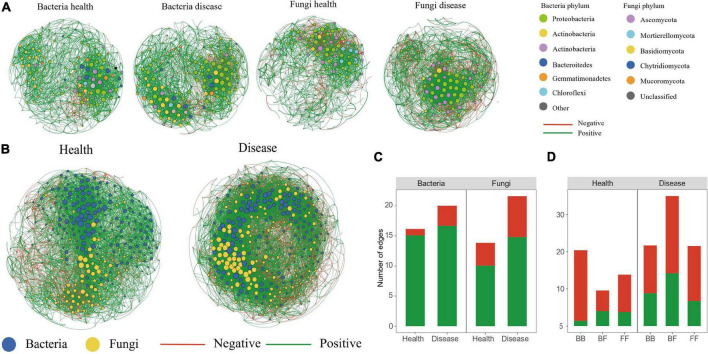
Co-occurrence networks. Intra-kingdom co-occurrence networks **(A)**. The nodes are colored according to bacterial phylum and fungal phylum. Node size indicates the degree of connection. Edge color represents positive (green) and negative (red) correlations. Number of positive and negative edges of bacterial and fungal groups in health and disease networks **(C)**. Interkingdom co-occurrence networks **(B)** contained both bacterial and fungal taxa. Number of bacterial–bacterial (BB), bacterial–fungal (BF), and fungal–fungal (FF) correlations in the healthy and diseased networks. Green and red colors of the edges and column indicate positive and negative correlations, respectively **(D)**.

To investigate how the disease affected the community structure of the peony microbiome, we performed a co-occurrence network analysis to explore the connection complexity within the rhizosphere microbiomes of both healthy and diseased soils (Figure 5). We also analyzed the bacteria–bacteria (BB), fungi–fungi (FF) intra-kingdom networks, along with the bacteria–fungi (BF) inter-kingdom networks. Based on the intra-kingdom network analysis (Figure 5A), we observed a higher proportion of negative edges and modularity in the fungal networks (proportion of negative edges/modularity: 27.2%/0.49 and 31.4%/0.41 in the healthy and diseased soils, respectively) than in the bacterial networks (proportion of negative edges/modularity: 0.67%/0.486 and 16.6%/0.495 in healthy and diseased soils, respectively). Furthermore, based on the number of nodes and edges, the network in diseased soil was more complex than in the healthy soil. The inter-kingdom co-occurrence networks further indicated that diseases destabilized the network and increased the intra-kingdom correlations among the fungal taxa (Figure 5C). The proportion of negative edges and modularity were higher in the diseased networks (proportion of negative edges/modularity: 38.2%/0.431) than in the healthy networks (proportion of negative edges/modularity: 21.1%/0.524) (Figure 5B). The BF inter-kingdom correlations were primarily negative (58.0% and 57.3% in the healthy and diseased networks, respectively), while negative correlations dominated the intra-kingdom correlations (93.0% BB and 72.8% FF in the healthy plants network, and 60.0% BB and 68.6% FF in the diseased network) (Figure 5D). The top-10 hub taxa were six bacteria and four fungi, and they were the same in both the healthy and diseased networks.

The topological roles of the nodes were defined by the within-module connectivity (Zi) and among-module connectivity (Pi). In all networks (Supplementary Figure 4), we found that 4.6% of the nodes were connectors (37 nodes), while 0.3% were module hubs (three nodes). Although we found no network hub in the four networks, the diseased network had more hubs (12 connectors in the bacterial network and nine connectors and one module hub in the fungal network) than the healthy network (eight connectors in the bacterial network and eight connectors and two module hubs in the fungal network). For the bacterial communities, the eight connectors in the healthy network belonged to *Lechevalieria, Stenotrophomonas, Rhodoplanes*, and *Nocardioides*, respectively. However, the 12 connectors in the healthy network belonged to *Reyranella, Sphingomonas*, and *Bradyrhizobium*, respectively. Regarding the fungal communities, *Solicoccozyma* and *Acremonium* were present in the diseased networks, while *Chaetomium* was in both the healthy and diseased networks.

## Discussion

### Effects of plant diseases on physical and chemical factors of soils

Microbes affect the growth of crops by affecting the soil’s physical and chemical properties, and consequently the vital transportation of nutrients needed for their cultivation (Jiang et al., 2017). The reduction in AK, AP, and OM decreased the soil quality and weakened plant resistance to pathogens (Janvier et al., 2007).

We analyzed the physicochemical properties of the rhizosphere soil of diseased and healthy plants in three areas. Compared to the healthy soils, the microorganisms in the root rot soil had higher carbon utilization ability, thereby potentially decreasing the OM content. The OM content and available nutrients were significantly decreased in the diseased soils, thus affecting the plant and soil microorganisms on soil nutrient utilization. Root rot actually increased the carbon source utilization capacity of plants, and the reduction of soil OM accelerated the occurrence of root rot, thus resulting in an inevitable vicious cycle that deepens the plants’ susceptibility.

### Changes in the microbial composition of diseased plants

This study presented no significant difference in bacteria and fungi between the diseased and healthy soils according to the phylum level’s diversity, richness index of the rhizosphere microorganisms, and OTUs. This result follows the results of previous studies (Jumpponen and Jones, 2010; Xu et al., 2012). We conducted further studies at the genus level, and upon comparing the diseased and healthy soils, we observed differences in the rhizosphere’s resident bacterial and fungal genera. These microbial genera may be the key genera to inhibit or aggravate the occurrence of root rot. In this study, we found abundant fungal OTUs belonging to *Fusarium*. The proportion of *Fusarium* in the diseased soils of D1, D2, and D3 increased by 3.38%, 1.15%, and 9.76%, respectively, as compared to healthy soils. Following our previous study results, *Fusarium* is the oil peony’s root rot pathogen (Liushuang, 2020). Moreover, sequencing results also showed that the number of *Fusarium* in the diseased soil was significantly higher than in the healthy soil. This study showed that the relative abundance of *Cylindrocarpon* and *Neocosmospora* in the fungal groups in diseased soil was significantly higher than in the healthy soil (Figure 3B). Furthermore, *Cylindrocarpon* and *Neocosmospora* were also the pathogenic genera in the soil of the root rot-infected plants. For example, *Cylindrocarpon* can cause peanut root rot (Caputo et al., 2015; Chojak-Koniewska et al., 2017). The relative abundance of *Chaetomium* is high in the healthy soil, and *Chaetomium* has been reported to produce antibiotics like chitin to inhibit the growth of pathogens, thus acting as an antagonistic fungus in the root rot soil (Pangesti et al., 2017). The significantly increased *Mycobacterium* and *Promicromonospora* in the soil of the root rot plants were bacteria related to OM degradation (Jin et al., 2018; Baranowski et al., 2019). *Dongia*, *Ohtaekwangia*, *Reyranella*, and other nutrient cycling-related bacteria also showed an increasing trend in the diseased plant soil (Piubeli et al., 2019; Sun et al., 2020; Zhang et al., 2020). The increase of these bacteria accelerated the degradation of soil nutrients, thus decreasing the soil nutrient content.

### Relationship between the available nutrient content and plant diseases

Soil microorganisms not only mutually affect each other but also interact with the surrounding environment. Increasing evidence indicated that root exudates initiate and modulate the dialog between the plants and soil microbes comprising both pathogenic and beneficial microbes (Zhao et al., 2016). Plants attract beneficial microbes by emitting volatile organic compounds or modifying the synthesis and secretion of particular root exudates (Berendsen et al., 2018; Jun et al., 2018; Liu et al., 2019; Gao et al., 2021). After planting oil peony for 5 years, the characteristics of the rhizosphere soil changes due to the shifting of the root exudates’ features because of the oil peony physiological change. This may systemically affect interactions between the plants and microbes *via* alteration of the microbial community structure. Therefore, our study aims to further understand the microbial activity mechanism and, through redundancy analysis, show a significant correlation between the soil properties and microbial community structure, which was affected by the OM and available nutrients in the diseased soil. Since the abundance of *Stenotrophomonas*, *Lysobacter*, *Nocardioides*, *Gaiella*, and *Sphingomonas* were negatively correlated with OM and the available nutrients, and root rot reduced the soil OM and the available nutrient content, we inferred that these bacteria might accelerate the soil’s carbon cycle. The abundance of *Marmoricolam* was positively correlated with both OM and the available nutrient content, and *Marmoricola* was also believed to improve the antifungal activity of the soil. Regarding fungi, redundancy analysis results indicated that the abundance of the *Fusarium* was negatively correlated with both OM and the available nutrients. Therefore, future studies can focus on controlling the content of the pathogenic *Fusarium* by manipulating the content of the organic compounds and available nutrients.

### The carbon metabolism of diseased plants increased

The BIOLOG ECO microplate technology can competently describe the metabolic functions of the microbial communities, especially for the environmental microorganisms (Choi and Dobbs, 1999). AWCD is related to the number and species of microbiota that utilize the single carbon source in a soil microbial community, thus reflecting their overall capacity to utilize the carbon source (Choi and Dobbs, 1999; De Gens et al., 2000). In this experiment, the AWCD of the soil microbes from the healthy and diseased plants exhibited the conventional microbial growth curve (from the adaptation to the stable phase), thereby presenting an increase during the culture time and being consistent with the previous studies (Kong et al., 2006; Wang et al., 2018). The growth curve showed that the higher the AWCD value, the higher the soil microbial metabolic activity (Wang et al., 2011). The diseased plants had higher AWCD values than the healthy plants. Thus, both the soil microbial activity and metabolic ability of the diseased plants were higher than the healthy plants. The pathogenic invasion of these plants altered the microbial community structure, which consequently increased their carbon source utilization, and this reduced the bioavailability of nutrients in plants, thereby aggravating the disease (Lan et al., 2019). In this study, the soil microbial metabolism enhancement may be related to the increase in specific microbial groups that can utilize carbohydrates and carboxylic acids. Additionally, plants release phenolic acids into the soil *via* aboveground leaching, root secretion, and plant residue decomposition. This directly affects the soil’s nutrient status, while indirectly affecting the plant growth by regulating soil microbial activity. In this study, the increase in metabolic capacity may also be due to the release of substances from the root post root rot infection and plant wilting, which causes more leaves to enter the soil, thus providing more carbon sources for soil microorganisms. The utilization rate analysis of the six carbon sources highlighted that the carbohydrate utilization ability of the soil microorganisms to in the root rot-infected oil peony root zone was significantly improved. This may be related to the increased abundance of related microbial taxa, e.g., *Dongia*, *Ohtaekwangia*, and *Reyranella*, in the root zone soil, which have been previously reported to degrade carbohydrates. Furthermore, this may be due to the root necrosis caused by root rot, which causes root organic components like sugars, organic acids, and amino acids to enter the soil, which may selectively increase the related microbial groups.

### Changes in microbial community structure of diseased plants

Cooperative and competitive interactions among the microbial species and network modularity can affect the community stability (Faust K; Coyte et al., 2015). In this study, fungal networks and their central taxa in healthy and diseased plants have more negative correlations than the bacterial networks. Mutual negative interactions indicate that ecological competition can reduce the stability of the microbial communities by inhibiting cooperation (Coyte et al., 2015). Therefore, the host may reduce its resistance to external stress due to microbial competition (Wagg et al., 2019). In stark contrast to bacterial communities, pathogen invasion affected the fungal communities more, probably due to enhanced positive intra-kingdom correlations we observed among the fungal taxa in diseased networks as compared to the healthy ones. Furthermore, lower modularity in the diseased fungal network may exacerbate the destabilizing effect due to the higher prevalence of cross-module correlations among the taxa (Grilli et al., 2016; Hernandez et al., 2021).

Many positive and negative correlations in the fungal network showed that the fungal community was more sensitive to diseased plants than the bacterial community.

Our results indicated that the diseased plants decreased the bacterial network complexity, but increased the fungal network complexity. The contrasting pattern of these networks parallels recent observation based on the macroecological soil patterns of *Fusarium* wilt (Yuan et al., 2020). Previous studies demonstrated the importance of the network complexity (Wagg et al., 2019) and hub taxa (Toju et al., 2018; Yu et al., 2020) in supporting the ecosystem functions. The fungal connectivity, mainly belonging to the intra-kingdom cooperative interactions, increased in the diseased soil, thus inducing the ecological importance of the fungal taxa. Additionally, we found dominating cooperative correlations within each microbial kingdom, but the competitive correlations dominated between the bacteria and fungi, since both the bacteria and fungi typically compete for similar plant-derived substrates (De et al., 2010).

Network hubs are the essential microorganisms in the microbial network (Qi et al., 2019). More network hubs, like keystone taxa, possibly made more frequent exchanges of materials and information among the microbial species in the diseased network than in the healthy network. Deciphering the network hub is critical for harnessing the plant microbiome to enhance the plant’s growth and health (Toju et al., 2018; Trivedi et al., 2020). Indeed, we found that several potential beneficial bacteria, like *Reyranella*, *Sphingomonas*, and *Bradyrhizobium*, were enriched in the diseased plants. We also identified them as the core taxa, i.e., present in all samples in the plant microbiomes. Nevertheless, previous studies showed that *Reyranella*, *Sphingomonas*, and *Bradyrhizobium* colonized plant rhizosphere and were vital for modulating the host performance, especially in suppressing plant pathogen (Zhang et al., 2020; Matsumoto et al., 2021). Therefore, our results indicated that the host plant might selectively regulate the community abundance of some core taxa under pathogen stress. Furthermore, we found that several fungal taxa, like *Solicoccozyma* and *Chaetomium*, were enriched in the diseased plants and identified as hub taxa in the co-occurrence networks, thereby having an antagonistic effect on the pathogens (Pangesti et al., 2017; Stosiek et al., 2019).

## Conclusion

This study highlighted the significant differences in the rhizosphere microbial community structures between the healthy and diseased oil peony plants. After pathogen invasion, the increase of bacteria related to nutrient cycling in soil, such as *Dongia, Ohtaekwangia, Reyranella*, etc., the decrease of antagonistic bacteria and fungi, like *Chaetomium, Marmoricola*, etc., and the increase of pathogenic fungi, like *Cylindrocarpon* and *Neocosmospora*, caused the imbalance of soil microbial community structure and eventually led to plant root rot. The soil’s physical and chemical properties changes and the modified carbon source utilization also indirectly indicated the changes in the rhizosphere microbial communities of the root rot plants. Moreover, the network’s diagram complexity and some parameters indicated that the rhizosphere microbial community had indeed changed. Given that oil peony is economically important and root rot has always been a serious concern, future studies can use our study results to screen antagonistic bacteria against root rot through the study of rhizosphere microorganisms of root rot plants. They can then use its interaction with other microorganisms in the network diagram to synthesize bacterial agents that can treat oil peony root rot, and also provide methods for biological control of oil peony root rot.

## Data availability statement

The data presented in this study are deposited in the NCBI (https://www.ncbi.nlm.nih.gov/) repository, accession numbers PRJNA850114 (16S) and PRJNA850456 (ITS).

## Author contributions

MJ, MC, SL, and JZ contributed to the conception and design of the study. SL and MJ performed a lot of experiments. MJ conducted statistical analysis and wrote the first draft of the manuscript. MC, XS, SL, and JZ wrote part of the manuscript. All authors contributed to the revision of the manuscript, read, and approved the submitted version.

## References

[B1] Baranowski C. Rego E. H. Rubin E. J. (2019). The dream of a mycobacterium. *Microbiol. Spectrum* 7. 10.1128/microbiolspec.GPP3-0008-2018 31025625 PMC11590427

[B2] Berendsen R. L. Vismans G. Yu K. Song Y. Jonge R. D. Burgman W. P. (2018). Disease-induced assemblage of a plant-beneficial bacterial consortium. *ISME J.* 12 1496–1507. 10.1038/s41396-018-0093-91 29520025 PMC5956071

[B3] Caputo F. Nicoletti F. Picione F. Manici L. M. (2015). Rhizospheric changes of fungal and bacterial communities in relation to soil health of multi-generation apple orchards. *Biol. Control* 88 8–17. 10.1016/j.biocontrol.2015.04.019

[B4] Cheffi M. Chenari Bouket A. Alenezi F. N. Luptakova L. Belka M. Vallat A. (2019). Olea europaea L. root endophyte bacillus velezensis OEE1 counteracts oomycete and fungal harmful pathogens and harbours a large repertoire of secreted and volatile metabolites and beneficial functional genes. *Microorganisms* 7:314. 10.3390/microorganisms7090314 31484434 PMC6780883

[B5] Choi K. H. Dobbs F. (1999). Comparison of two kinds of Biolog microplates (GN and ECO) in their ability to distinguish among aquatic microbial communities. *J. Microbiol. Methods* 36 203–213. 10.1016/s0167-7012(99)00034-2 10379806

[B6] Chojak-Koniewska J. Linkiewicz A. Sowa S. Radzioch M. A. Kuniak E. (2017). Interactive effects of salt stress and *Pseudomonas syringae* pv. lachrymans infection in cucumber: involvement of antioxidant enzymes, abscisic acid and salicylic acid. *Environ. Exp. Botany* 136 9–20. 10.1016/j.envexpbot.2017.01.004

[B7] Coyte K. Z. Schluter J. Foster K. R. (2015). The ecology of the microbiome: networks, competition, and stability. *Science* 350 663–666. 10.1126/science.aad2602 26542567

[B8] De B. W. Folman L. B. Summerbell R. C. Lynne B. (2010). Living in a fungal world: impact of fungi on soil bacterial niche development. *FEMS Microbiol. Rev.* 29 795–811. 10.1016/j.femsre.2004.11.005 16102603

[B9] De V. Griffiths R. I. Mark B. Hayley C. Mariangela G. Soon G. H. (2018). Soil bacterial networks are less stable under drought than fungal networks. *Nat. Commun.* 9:3033. 10.1038/s41467-018-05516-5517 PMC6072794 30072764

[B10] De Gens B. P. Schipper L. A. Sparling G. P. Vojvodic-Vukovic M. (2000). Decreases in organic C reserves in soils can reduce the catabolic diversity of soil microbial communities. *Soil Biol. Biochem.* 32 189–196. 10.1016/S0038-0717(99)00141-148

[B11] Dugan F. M. Hellier B. C. Lupien S. L. (2010). First report of *Fusarium proliferatum* causing rot of garlic bulbs in North America. *Plant Pathol.* 52 426–426. 10.1046/j.1365-3059.2003.00852.x

[B12] Duran P. Thiergart T. Garrido-Oter R. Agler M. Kemen E. Schulze-Lefert P. (2018). Microbial interkingdom interactions in roots promote arabidopsis survival. *Cell* 175 973–983.e14. 10.1016/j.cell.2018.10.020. 30388454 PMC6218654

[B13] Faoro H. Alves A. C. Souza E. M. Rigo L. U. Cruz L. M. Al-Janabi S. M. (2010). Influence of soil characteristics on the diversity of bacteria in the Southern brazilian atlantic forest. *Appl. Environ. Microb* 76 4744–4749. 10.1128/AEM.03025-3029 PMC2901723 20495051

[B14] Gao M. Xiong C. Gao C. Tsui C. K. M. Wang M. M. Zhou X. (2021). Disease-induced changes in plant microbiome assembly and functional adaptation. *Microbiome* 9:187. 10.1186/s40168-021-01138-1132 PMC8444440 34526096

[B15] Grilli J. Rogers T. Allesina S. (2016). Modularity and stability in ecological communities. *Nat. Commun.* 7:12031. 10.1038/ncomms12031 27337386 PMC4931019

[B16] Hernandez D. J. David A. S. Menges E. S. Searcy C. A. Afkhami M. E. (2021). Environmental stress destabilizes microbial networks. *ISME J.* 15 1722–1734. 10.1038/s41396-020-00 33452480 PMC8163744

[B17] Hooks K. B. O’Malley M. Davies J. E. (2017). Dysbiosis and its discontents. *mBio* 8:e01492-17. 10.1128/mBio.01492-1417 PMC5635691 29018121

[B18] Inclán R. Daniel D. L. T. Benito M. Rubio A. (2015). Soil CO2 efflux in a mixed pine-oak forest in Valsaín (central Spain). *Sci. World J.* 7 166–174. 10.1100/tsw.2007.7 17450294 PMC5901050

[B19] Jaffuel G. Mäder P. Blanco-Perez R. Chiriboga X. Fliessbach A. Turlings T. (2016). Prevalence and activity of entomopathogenic nematodes and their antagonists in soils that are subject to different agricultural practices. *Agriculture Ecosystems Environ.* 230 329–340. 10.1016/j.agee.2016.06.009

[B20] Janvier C. Villeneuve F. Alabouvette C. Edel-Hermann V. Mateille T. Steinberg C. (2007). Soil health through soil disease suppression: which strategy from descriptors to indicators? *Soil Biol. Biochem.* 39 1–23. 10.1016/j.soilbio.2006.07.001

[B21] Jiang J. Song Z. Yang X. Mao Z. Nie X. Guo H. (2017). Microbial community analysis of apple rhizosphere around Bohai Gulf. *Sci. Rep.* 7:8918. 10.1038/s41598-017-08398-8399 PMC5566992 28827532

[B22] Jin L. Zhao J. Jiang S. Zhao Y. Han X. Guo X. (2018). *Promicromonospora viridis* sp. nov., a novel actinomycete isolated from soil. *Antonie Van Leeuwenhoek* 111 2079–2086. 10.1007/s10482-018-1099-1094 29779147

[B23] Jumpponen A. Jones K. L. (2010). Seasonally dynamic fungal communities in the *Quercus macrocarpa* phyllosphere differ between urban and nonurban environments. *New Phytol.* 186 496–513. 10.1111/j.1469-8137.2010.03197.x 20180911

[B24] Jun Y. Jun Z. Tao W. Mengli Z. Rong L. Pim G. (2018). Root exudates drive the soil-borne legacy of aboveground pathogen infection. *Microbiome* 6:156. 10.1186/s40168-018-0537-x 30208962 PMC6136170

[B25] Kendrick B. (2003). Ainsworth & bisby’s dictionary of the fungi. *Mycologist* 17 17–19. 10.1079/9780851998268.0000

[B26] Kong W. D. Zhu Y. G. Fu B. J. Marschner P. He J. Z. (2006). The veterinary antibiotic oxytetracycline and Cu influence functional diversity of the soil microbial community. *Environ. Pollut.* 143 129–137. 10.1016/j.envpol.2005.11.003 16413090

[B27] Lan X. Du H. Peng W. Liu Y. Song T. (2019). Functional diversity of the soil culturable microbial community in eucalyptus plantations of different ages in guangxi, South China. *Forests* 10:1083. 10.3390/f10121083

[B28] Langfelder P. Horvath S. (2007). Eigengene networks for studying the relationships between co-expression modules. *BMC Systems Biol.* 1:54. 10.1186/1752-0509-1-54 18031580 PMC2267703

[B29] Lauber C. L. Strickland M. S. Bradford M. A. Fierer N. (2008). The influence of soil properties on the structure of bacterial and fungal communities across land-use types. *Soil Biol. Biochem.* 40 2407–2415. 10.1016/j.soilbio.2008.05.021

[B30] Li C. Li X. Kong W. Wu Y. Wang J. (2010). Effect of monoculture soybean on soil microbial community in the Northeast China. *Plant Soil* 330 423–433. 10.1007/s11104-009-0216-216

[B31] Liu H. Macdonald C. A. Cook J. Anderson I. C. Singh B. K. (2019). An ecological loop: host microbiomes across multitrophic interactions. *Trends Ecol. Evol.* 34 1118–1130. 10.1016/j.tree.2019.07.011 31422890

[B32] Liushuang. (2020). *Study on Rhizosphere Microecology Characteristics and Biological Control of Paeonia Suffruticosa Root rot in Shanxi, China.* Master’s degree thesis, China: Beijing Forestry University.

[B33] Matsumoto H. Fan X. Wang Y. Kusstatscher P. Wang M. (2021). Bacterial seed endophyte shapes disease resistance in rice. *Nat. Plants* 7 60–72. 10.1038/s41477-020-00826-825 33398157

[B34] Mueller K. E. Eisenhauer N. Reich P. B. Hobbie S. E. Oleksyn J. (2016). Light, earthworms, and soil resources as predictors of diversity of 10 soil invertebrate groups across monocultures of 14 tree species. *Soil Biol. Biochem.* 92 184–198. 10.1016/j.soilbio.2015.10.010

[B35] Ning L. Huang Q. Guo S. Shen Q. (2011). *Paenibacillus polymyxa* SQR-21 systemically affects root exudates of watermelon to decrease the conidial germination of *Fusarium oxysporum* f.sp. niveum. *Plant Soil* 341 485–493. 10.1007/s11104-010-0660-663

[B36] Pangesti N. Vandenbrande S. Pineda A. Dicke M. Raaijmakers J. M. Loon J. J. A. V. (2017). Antagonism between two root-associated beneficial Pseudomonas strains does not affect plant growth promotion and induced resistance against a leaf-chewing herbivore. *FEMS Microbiol. Ecol.* 93. 10.1093/femsec/fix038 28334335

[B37] Piubeli F. A. Piubeli F. A. Gibbi L. Santos D. Fernández E. N. Flávio H. (2019). The emergence of different functionally equivalent PAH degrading microbial communities from a single soil in liquid PAH enrichment cultures and soil microcosms receiving PAHs with and without bioaugmentation. *Polish J. Microbiol.* 67 365–375. 10.21307/pjm-2018-046 30451454 PMC7256725

[B38] Qi G. Ma G. Chen S. Lin C. Zhao X. (2019). Microbial network and soil properties are changed in bacterial wilt-susceptible soil. *Appl. Environ. Microbiol.* 85:e00162-19. 10.1128/AEM.00162-119 PMC6581179 31003986

[B39] Restrepo O. Flores J. Arboleda F. M. (2015). Influence of management systems on the nitrogen mineralization and fertilization of sugarcane. *Rev. Facultad Nacional Agronoma Medellin* 69 7755–7762. 10.15446/rfna.v69n1.54742

[B40] Rousk J. Bååth E. Brookes P. C. Lauber C. L. Lozupone C. Caporaso J. G. (2010). Soil bacterial and fungal communities across a pH gradient in an arable soil. *ISME J.* 4 1340–1351. 10.1038/ismej.2010.58 20445636

[B41] Stosiek N. Terebieniec A. Zbek A. Mynarz P. Cieliński H. Klimek-Ochab M. (2019). N-phosphonomethylglycine utilization by the psychrotolerant yeast Solicoccozyma terricola M 3.1.4. *Bioorgan. Chem.* 93:102866. 10.1016/j.bioorg.2019.03.040 30902434

[B42] Sun J. Yang L. Wei J. Quan J. Yang X. (2020). The responses of soil bacterial communities and enzyme activities to the edaphic properties of coal mining areas in Central China. *PLoS One* 15:e0231198. 10.1371/journal.pone.0231198 32343698 PMC7188301

[B43] Toju H. Peay K. G. Yamamichi M. Narisawa K. Hiruma K. Naito K. (2018). Core microbiomes for sustainable agroecosystems. *Nat. Plants* 4 247–257. 10.1038/s41477-018-0139-134 29725101

[B44] Trivedi P. Leach J. E. Tringe S. G. Sa T. Singh B. K. (2020). Plant-microbiome interactions: from community assembly to plant health. *Nat. Rev. Microbiol.* 18 607–621. 10.1038/s41579-020-0412-411 32788714

[B45] van der Heijden M. G. de Bruin S. Luckerhoff L. van Logtestijn R. S. Schlaeppi K. (2016). A widespread plant-fungal-bacterial symbiosis promotes plant biodiversity, plant nutrition and seedling recruitment. *ISME J.* 10 389–399. 10.1038/ismej.2015.120 26172208 PMC4737930

[B46] Wagg C. Schlaeppi K. Banerjee S. Kuramae E. E. Heijden M. (2019). Fungal-bacterial diversity and microbiome complexity predict ecosystem functioning. *Nat. Commun.* 10:4841. 10.1038/s41467-019-12798-y 31649246 PMC6813331

[B47] Wang C. Xue L. Dong Y. Wei Y. Jiao R. (2018). Unravelling the functional diversity of the soil microbial community of chinese fir plantations of different densities. *Forests* 9:532. 10.3390/f9090532

[B48] Wang J. Xu C. Sun Q. Xu J. Chen Y. (2021). Post-translational regulation of autophagy is involved in intra-microbiome suppression of fungal pathogens. *Microbiome* 9:131. 10.1186/s40168-021-01077-y 34092253 PMC8182927

[B49] Wang Y. Ouyang Z. Zheng H. Wang X. Chen F. Zeng J. (2011). Carbon metabolism of soil microbial communities of restored forests in Southern China. *J. Soils Sediments Protection Risk Assess. Rem* 11 789–799. 10.1007/s11368-011-0352-355

[B50] Weller D. M. Raaijmakers J. M. Gardener B. B. Thomashow L. S. (2002). Microbial populations responsible for specific soil suppressiveness to plant pathogens. *Annu. Rev. Phytopathol.* 40 309–309. 10.1146/annurev.phyto.40.030402.110010 12147763

[B51] Wu L. Wang J. Huang W. Wu H. Chen J. Yang Y. (2016). Plant-microbe rhizosphere interactions mediated by *Rehmannia glutinosa* root exudates under consecutive monoculture. *Sci. Rep.* 5:15871. 10.1038/srep15871 26515244 PMC4626807

[B52] Xia Z. Bai E. Wang Q. Gao D. Zhou J. Jiang P. (2016). Biogeographic distribution patterns of bacteria in typical chinese forest soils. *Front. Microbiol.* 7:1106. 10.3389/fmicb.2016.01106 27468285 PMC4942481

[B53] Xu L. Ravnskov S. Larsen J. Nilsson R. H. Nicolaisen M. (2012). Soil fungal community structure along a soil health gradient in pea fields examined using deep amplicon sequencing. *Soil Biol. Biochem.* 46 26–32. 10.1016/j.soilbio.2011.11.010

[B54] Yu S. A. Db B. Yl A. Yy C. Ygzd E. Jpf G. (2020). Abundance of kinless hubs within soil microbial networks are associated with high functional potential in agricultural ecosystems. *Environ. Int.* 142:105869. 10.1016/j.envint.2020.105869 32593837

[B55] Yuan J. Raza W. Shen Q. Huang Q. (2012). Antifungal activity of bacillus amyloliquefaciens NJN-6 volatile compounds against *Fusarium oxysporum* f. sp. cubense. *Appl. Environ. Microbiol.* 78 5942–5944. 10.1128/AEM.01357-1312 22685147 PMC3406121

[B56] Yuan J. Wen T. Zhang H. Zhao M. Shen Q. (2020). Predicting disease occurrence with high accuracy based on soil macroecological patterns of Fusarium wilt. *ISME J.* 14 2936–2950. 10.1038/s41396-020-0720-725 32681158 PMC7784920

[B57] Zhang P. Huang P. Xu X. Sun H. Jiang B. Liao Y. (2020). Spectroscopic and molecular characterization of biochar-derived dissolved organic matter and the associations with soil microbial responses. *Sci. Total Environ.* 708:134619. 10.1016/j.scitotenv.2019.134619 31791751

[B58] Zhao Y. P. Lin S. Chu L. Gao J. T. Azeem S. Lin W. (2016). Insight into structure dynamics of soil microbiota mediated by the richness of replanted *Pseudostellaria heterophylla*. *Sci. Rep.* 6:26175. 10.1038/srep26175 27188449 PMC4870612

