# Supplementary material for: Deciphering the microbial diversity associated with healthy and wilted Paeonia suffruticosa rhizosphere soil
Source: Front Microbiol. 2022 Aug 17;13:967601. doi: 10.3389/fmicb.2022.967601 (PMC9432862; doi:10.3389/fmicb.2022.967601)
Supplement: Supplementary file 1 [file Image_1.PDF]

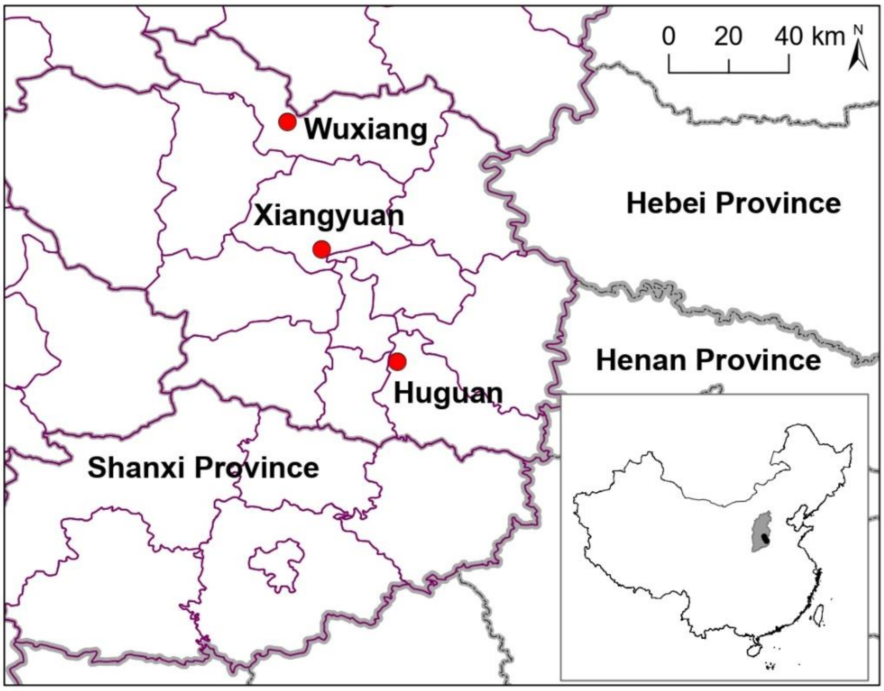

Fig.S1 Location of sampling

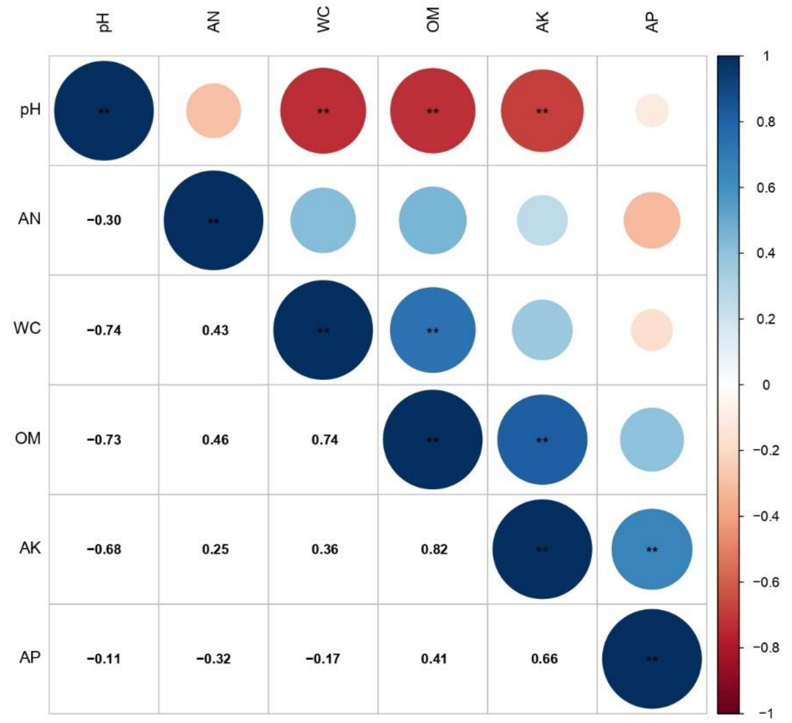

Fig.S2. Environmental factor. AN is available nitrogen, AK is available potassium, AP is available phosphorus, WC is moisture content, OM is organic matter

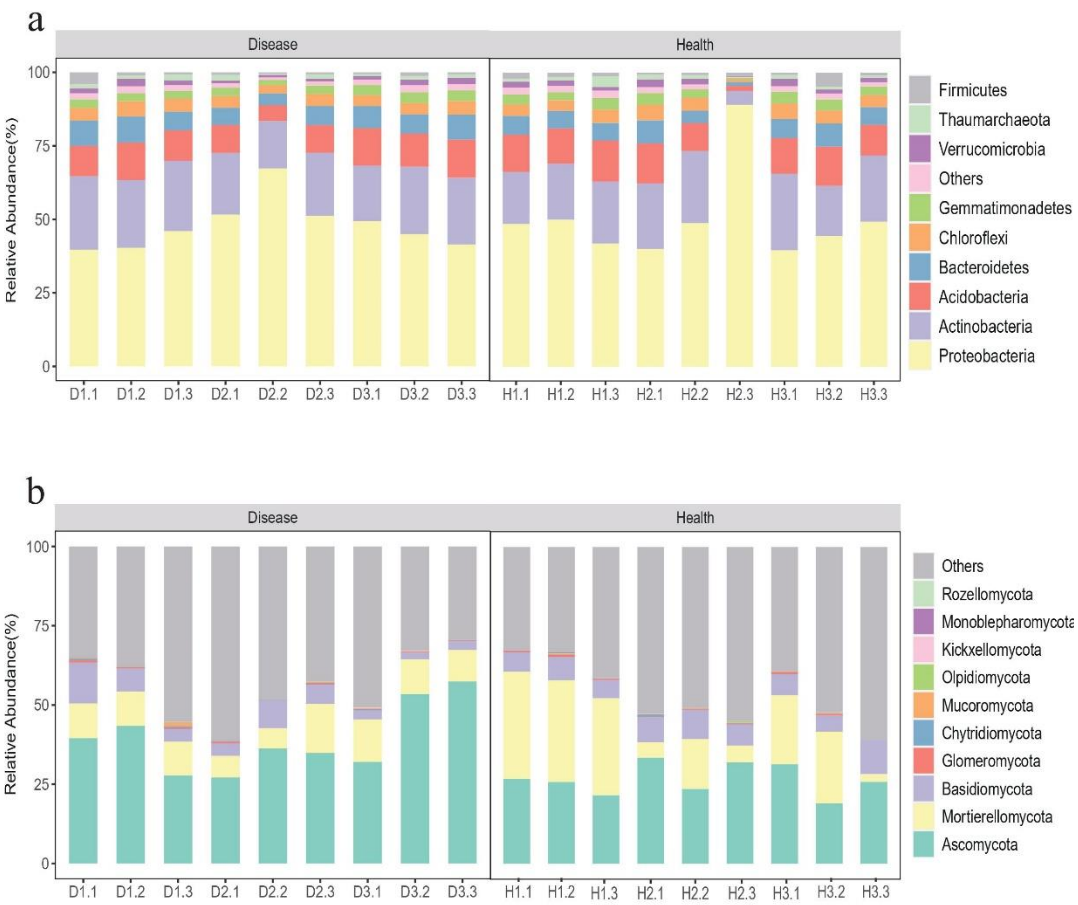

Fig. S3. Horizontal abundance of microbial phylum. The average relative abundances of bacterial (a) and fungal (b) phylum from diseased and healthy plant soils. Others include phylum below 0.1% of relative abundance and the unclassified phylum. The“H1”, “H2” and“H3” refer to the three healthy soils. The“D1”, “D2” and“D3” refer to the three FRR soils, respectively.

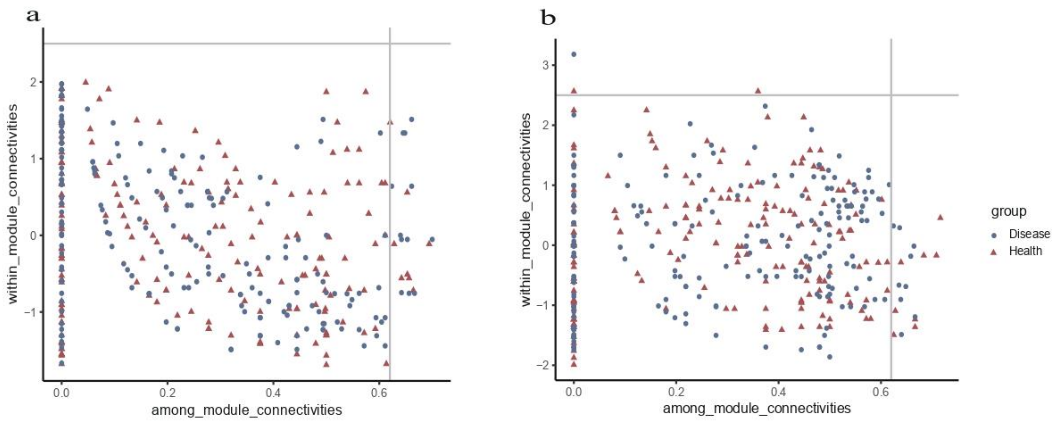

Fig. S4. Topological roles of nodes. Node topological properties of bacterial (a) and (b) fungal networks. Threshold values of Zi and Pi for categorizing nodes are 2.5 and 0.62.
